# The spectrum of clinical presentation in haploinsufficiency of A20; a case report of a novel mutation in *TNFAIP3* gene

**DOI:** 10.3389/fped.2023.1132596

**Published:** 2023-06-14

**Authors:** M. Debeljak, S. Blazina, J. Brecelj, T. Avčin, N. Toplak

**Affiliations:** ^1^ Clinical Institute for Special Laboratory Diagnostics, University Children’s Hospital Ljubljana, University Medical Centre Ljubljana, Ljubljana, Slovenia; ^2^ Faculty of Medicine, University of Ljubljana, Ljubljana, Slovenia; ^3^ Department of Allergology, Rheumatology and Clinical Immunology, University Children’s Hospital, University Medical Centre Ljubljana, Ljubljana, Slovenia; ^4^ Department of Gastroenterology, Hepatology and Nutrition, University Children’s Hospital Ljubljana, University Medical Centre Ljubljana, Ljubljana, Slovenia

**Keywords:** haploinsufficiency A20, TNFAIP3 gene, NGS genetic testing, case report, clinical presentation

## Abstract

Haploinsufficiency of A20 was first described in 2016 as a new autoinflammatory disease that clinically presents as early-onset Behcet's disease. After the publication of the first 16 cases, more patients were diagnosed and described in the literature. The spectrum of clinical presentation has expanded. In this short report, we present a patient with a novel mutation in the *TNFAIP3* gene. The clinical presentation included signs of an autoinflammatory disease with recurrent fever, abdominal pain, diarrhea, respiratory tract infections, and elevated inflammatory parameters. We will emphasize the importance of genetic testing, especially in patients with various clinical signs that do not fit a single autoinflammatory disease.

## 1. Introduction

Haploinsufficiency A20 (HA20) is a novel autoinflammatory disease first described in 2016 and belongs to the group of diseases with disturbance of the ubiquitination process (1, 2). The gene *TNFAIP3* (6q23.3) has 9 exons. It codes for A20 protein, also called TNF*α* Induced Protein 3 (TNF/AIP3), which has a negative regulatory role in inflammation. A20 protein is a cytoplasmic zinc finger protein that restricts NF*κ*B signals via deubiquitinating activity (1). Loss-of-function mutations in *TNFAIP3* result in the increased degradation of *κ*B inhibitor, which leads to activation of the nuclear factor (*N*F)-*κ*B pathway, an increased expression of proinflammatory cytokines, and systemic inflammation (3).

Patients may present with early-onset systemic inflammation and a Behcet-like disease, or a variety of autoinflammatory and autoimmune features (4).

Haploinsufficiency A20 is an autosomal dominant disorder with heterozygous truncating mutations in the *TNFAIP3* gene. Currently, there are 75 variants described in the Infever database (5).

We report a novel mutation that resulted in a stop codon in exon 7. Clinical presentation included various clinical signs. We will emphasize the importance of genetic testing of a panel of inflammatory genes in case of an unclear clinical picture.

## 2. Case presentation

A 14-year-old patient was first examined at the Immunology/Rheumatology outpatient office at the age of 9 because of recurrent fever with diarrhea, abdominal pain, elevated inflammatory markers, and recurrent respiratory infections. Aphthae in the mouth appeared a few times from the age of 1 up to the age of 8 and after that disappeared. He never had genital aphthae.

Family history was unremarkable except for migraine in the father and mother.

The child was born after the mother's first pregnancy. Prenatal ultrasound (US) showed hypo echogenic bowel and short femur. Karyotype was normal. Mother had gestational diabetes which improved with diet. Due to intrauterine growth retardation, labor was induced prematurely, at 33 weeks of gestation. Birth weight was 1.350 kg, the Apgar score was 8/8, and the postpartum course was unremarkable.

Fevers started at 7 weeks of age and were mainly triggered by infections. In several episodes, the fever was accompanied by vomiting. He was constantly having a runny nose. In the first 2 months of life, he was fed normally. In the third month, the parents noticed mucus in the stool, bloating, abdominal cramps, and signs of gastroesophageal reflux. From 18 to 22 months of age, he had recurrent respiratory infections.

From the age of 2.5 years, episodic attacks of severe abdominal pain with vomiting started. Episodes lasted 3–5 days and were accompanied by respiratory infections and fever. The child was followed in the gastroenterology, allergology, and pulmonology outpatient offices. He was tested for food allergy and was found to be low positive for milk and egg IgE. Because of respiratory infections and digestive problems, he was tested for cystic fibrosis. Investigations, including the genetic testing, were negative.

The parents never noticed rashes or arthritis but he had muscle pains. Parents noticed that the child's problems were aggravated by the cold.

At the age of 7, he was admitted to the surgical department due to abdominal pain, fever, and vomiting. Intussusception was found and was resolved by hydro-colon. CRP was 113 mg/L (*N* < 8 mg/L). After that episode, he had severe abdominal pain several times. Serology for celiac disease was normal. Fecal calprotectin was mildly elevated (around 120 mg/kg) several times so upper and lower gastrointestinal (GIT) endoscopy was performed. Except for pronounced lymphatic hypertrophy in the terminal ileum, the investigation was normal. Histology revealed non-specific mild inflammatory changes. Infections were excluded. Abdominal ultrasound and magnetic resonance enterography did not show any signs of inflammation. He continued to have attacks of abdominal pain until a diagnosis was made and specific treatment started.

**Figure 1:**
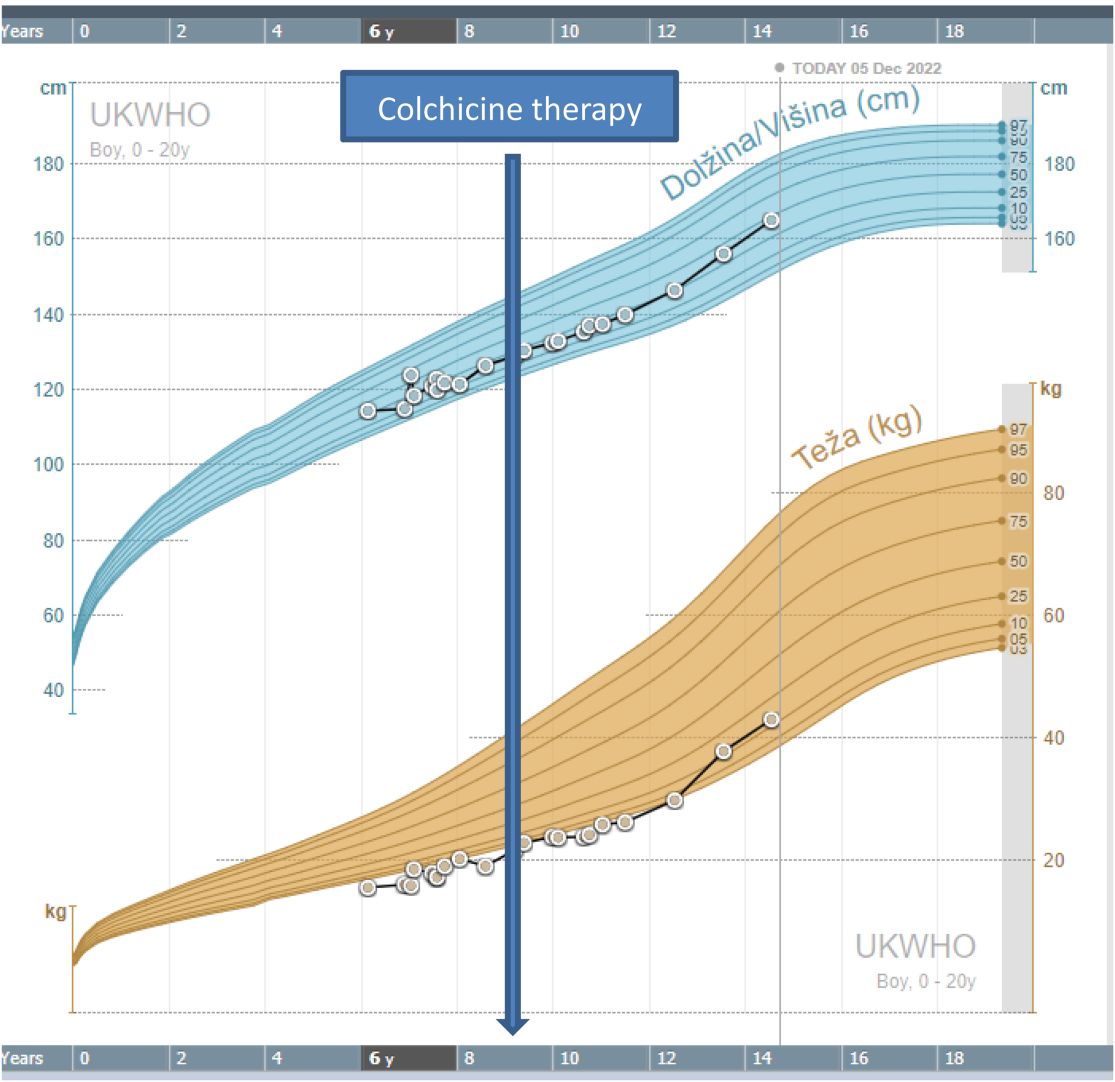
Growth chart of the patient.

At the age of 9, he was referred for the first time for an immunological evaluation due to suspected immunodeficiency. His growth and weight were normal although on the lower percentiles (Figure 1). Nitroblue tetrazolium test (NBT), for exclusion of chronic granulomatous disease, was normal. Serum immunoglobulin levels IgG, IgA, and IgE were elevated- IgG 17.9 g/L (*N* 4.62–16.82), IgA 4.06 g/L (N 0.34–2.74), and IgE 115 IU/ml (*N* < 90), whereas serum level of IgM was normal-0.8 g/L (N 0.38–2.51). Flow cytometry showed a decreased concentration of helper T cells (CD4+) and cytotoxic T cells (CD8+) at the age of 9 and normal concentrations at the age of 10.5 (Table 1). The concentration of recent thymic emigrants (RTE) was normal. T cells differentiation showed an increased proportion of double negative T cells (*α*/*β* and *γ*/*δ* TCR + CD3 + CD45 + CD4-CD8-) (Table 2). Antinuclear antigen-antibody (ANA- Hep2) was negative.

**Table 1:** Flow cytometry.

	CD19+	CD3+	CD3 + CD4+	CD3 + CD8+	NK	HLA-DR
C	0.246 × 10^9^	0.754 × 10^9^	0.371 × 10^9^	0,289 × 10^9^	0.180 × 10^9^	0.151 × 10^9^
Age 9	(*N* 0.3–0.7)	(*N* 1.1–2.8)	(*N* 0.5–1.8)	(*N* 0.4–1.2)	(*N* 0.1–0.6)	(*N* 0.08–0.5)
C	0.274 × 10^9^	1.435 × 10^9^	0.511 × 10^9^	0.431x × 10^9^	0.268	0.703
Age 10.5	(*N* 0.2–0.5)	(*N* 1.0–2.0)	(*N* 0.5–1.3)	(*N* 0.3–0.8)	(*N* 0.1–0.7)	(*N* 0.03–0.2)

**Table 2:** T cell differentiation.

	CD4 + CD45RA	CD4 RTE	^a^TCR*α*/*β* DNT (CD4-CD8-)	^a^TCR*γ*/*δ* DNT (CD4-CD8-)	cIFN*γ* + CD4+
C / %	C 0.261 × 10^9^	C 0.194 × 10^9^	5% (*N* < 4%)	37% (*N* < 5%)	19% (*N*10–26%)
Age 10.5	(*N* 0.24–0.7)				
		(*N* 0.05–0.926)			

C-concentration (cells/l).

^a^ DNT cells are CD3 + and CD45+; % of DNT is calculated among CD3 + cells.

After all investigations, it was concluded that the most probable diagnosis was immune dysregulation with autoinflammation, resembling one of the periodic fever syndromes. The blood was sent for genetic testing.

## 3. Genetic testing

Due to atypical clinical presentation with signs of autoinflammation not specific to a single disease, Next-Generation Sequencing (NGS) was performed.

A whole blood EDTA sample was used for the extraction of genomic DNA according to established laboratory protocols using a FlexiGene DNA isolation kit (Qiagen, Germany). NGS sequencing was performed using a MiSeq desktop sequencer coupled with MiSeq Reagent kit v3 (both Illumina, San Diego, USA). The regions of interest were enriched using the TruSightOne library enrichment kit (Illumina, San Diego, USA) following the manufacturer's instructions. The sequencing data reached 99.9% for at least 10X coverage for a patient. The selected core panel of autoinflamatory genes (*CARD14, ELANE, HAX1, IL10, IL10RA, IL10RB, IL1RN, IL36RN, LPIN2, MEFV, MVK, NLRP12, NLRP3, NOD2, NLRP7, PLCG2, PSTPIP1, SH3BP2, SLC29A3, TMEM173, TNFAIP3, TNFRSF11A, and TNFRSF1A)* was used for filtering.

In the *TNFAIP3* gene, which codes for Tumor Necrosis Factor-Alpha-Induced Protein 3, the heterozygous substitution NM_006290.3:c.1084C > T was found. It changes amino acid glutamine at position 362 into termination codon (NP_006281.1:p.Gln362Ter). Nomenclature is cited according to the HGVS guidelines (www.hgvs.org/mutnomen). The potential deleterious effect of identified genetic variants was analyzed with *in silico* prediction tools: CADD score: Pherd 36 (http://cadd.gs.washington.edu/score) and Mutation taster: disease-causing 0,99 (http://www.mutationtaster.org/). The variant was not present in dbSNP or gnomAD database (genome Aggregation Database: https://gnomad.broadinstitute.org/).

The variant was confirmed with Sanger sequencing (ABI Genetic Analyzer 3500, Applied Biosystems, USA). Family segregation analysis was performed in both parents in whom identified mutation was not present.

The mutation in the patient was *de novo* and novel in a spectrum of mutations for A20 Haploinsufficiency. Genetic testing was also performed on the parents. No mutations in the *TNFAIP3* gene were found.

## 4. Treatment and follow-up

**Figure 2:**
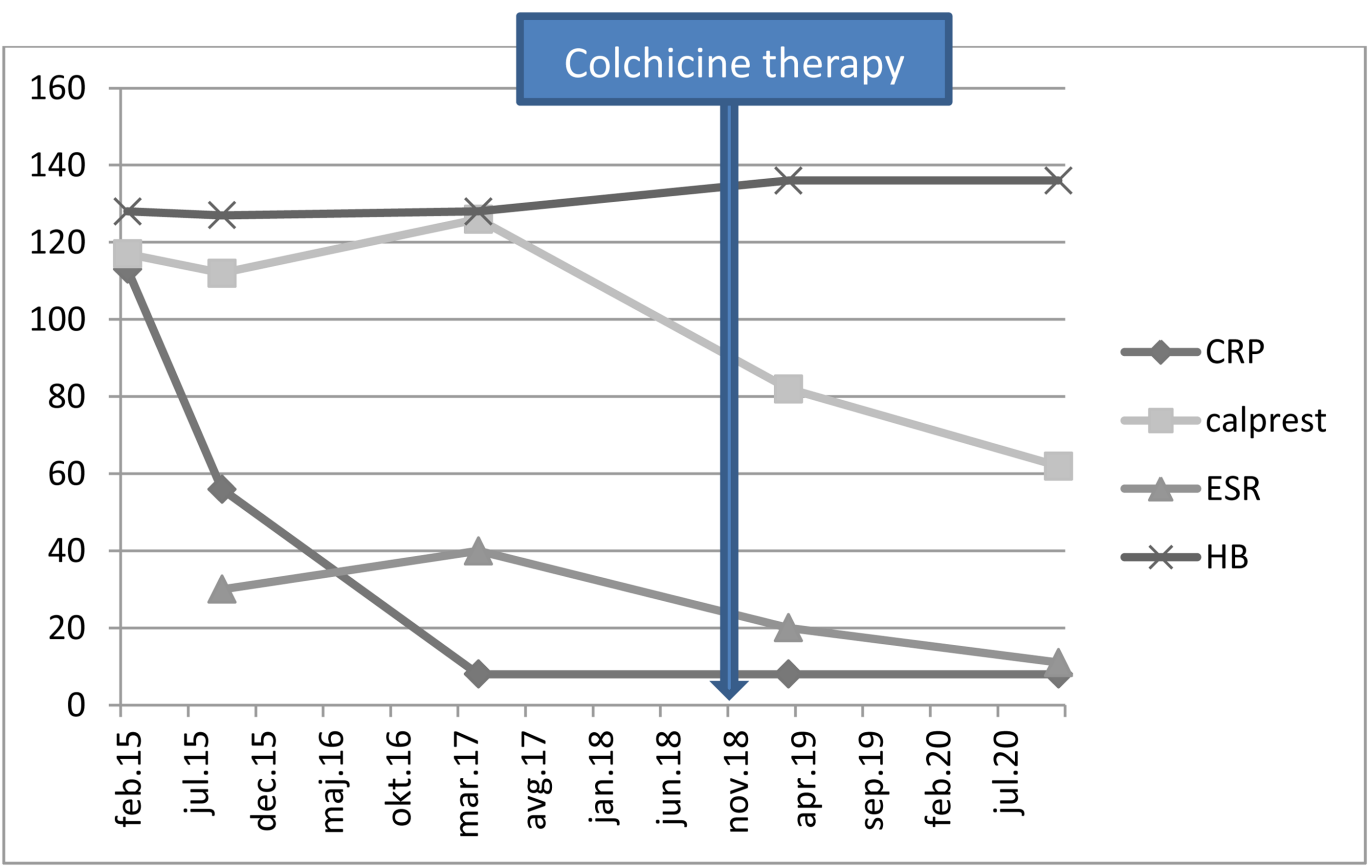
Laboratory results of the patient. CRP- C-reactive protein (mg/L); Calprest- calprotectin (mg/kg); ESR- erythrocyte sedimentation rate (mm/h); HB- hemoglobin level (g/L).

After the result of genetic testing, in November 2018, the child started colchicine therapy at a dose of 0.5 mg once per day. In January 2019, the dose was increased to 0.5 mg twice per day. The attacks of abdominal pains and fever stopped and the child became stable and has been growing normally, according to his growth percentile. The inflammatory parameters and stool calprotectin were normalized with therapy (Figure 2).

## 5. Discussion

We described a patient with a novel mutation in the *TNFAIP3* gene who presented with a milder clinical presentation with recurrent respiratory infections that are common in early childhood. He was having abdominal pains, recurrent diarrhea, and fever attacks since early infancy, from the age of 2 years old. His growth and development were within normal limits. He had few aphthae from the age of 1 to 8, no arthritis, and no rash. In the first published cohort of 16 patients from seven unrelated families, all patients had recurrent oral, genital, and/or GIT ulcers. Musculoskeletal complaints were present in 9/16, GIT complaints in 9/16, cutaneous lesions in 8/16, episodic fever in 7/16, and recurrent infections in 7/16. ANA was found in 4/10, and anti-DNA in 2/5 patients who were tested. A biopsy of the gut showed non-specific chronic inflammation (4). Our patient had five characteristics from the abovementioned list, namely, oral aphthae up to the age of 8, GIT complaint, episodic fever, recurrent infections, and non-specific chronic inflammation on gut biopsy. Recently, Yu et al. published an analysis of all cases reported so far (6). Together they found 61 patients from 26 families, 62% were women. Patients had highly variable clinical manifestations and had been diagnosed with several diseases including Behcet's disease, Rheumatoid arthritis, Rheumatic fever, Juvenile idiopathic arthritis, Periodic fever syndrome with pharyngitis, adenitis and aphthous changes, Crohn's disease, Systemic lupus erythematosus, and even adult-onset Stills' disease. Initial symptoms occurred early, at a mean age of 14 (5 d to 29 y), 73% of patients (*n *= 45) experienced their first symptoms before the age of 10, 64% of patients reported oral and/or genital ulcers, only five patients had ocular manifestations, which would be typical for Behcet's, 44% had a fever, 44% had GIT complaints, 43% skin changes, and 33% musculoskeletal complaints. Our patient clinically fits in this group.

Because fever episodes were almost always accompanied by respiratory infections he was tested for suspected immune deficiency but the results were not suggesting the inborn error of humoral and cellular immunity. The most remarkable finding was an increased proportion of *α*/*β* TCR + double negative T cells that have been implicated in the pathogenesis of autoimmune and autoinflammatory diseases (7). The disease from the spectrum of immune dysregulation was suspected and the blood was sent for genetic testing. In a recently published case report of a 3-year-old and a 6-month-old child with HA20, the percentages of CD19+, CD3+, CD3 + CD4+, CD3 + CD8+, and NK cells were normal, however, the concentrations were not presented (8). Interestingly, the concentration of serum IgG was elevated as it was also in our patient.

Our patient was treated with colchicine, which is described as a successful drug in alleviating attacks in number and severity. In a recent review, nearly half of the patients responded to colchicine treatment (6). Our patient even had an acceleration of growth after colchicine was introduced (Figure 1). Some patients were also successfully treated with other immunomodulatory drugs, including glucocorticosteroids, mesalazine, cyclosporine, methotrexate, and azathioprine. Anti-cytokine therapy with anakinra, rituximab, tocilizumab, and infliximab has also been used in some patients (6). A recent report pointed to Janus kinase (JAK) inhibitor as a potential successful therapy for HA20. Schwartz et al. reported that a type I interferon signature, or elevation of IFN- stimulated genes correlated with disease activity and predicted response to JAK inhibition in HA20 (9). In this study, the spectrum of HA20-associated phenotypes was also extended to include severe hepatic inflammation in the absence of systemic features.

In our patient, a novel and *de novo* heterozygous truncating mutation in the *TNFAIP3* gene (p.Gln362Ter), which causes haploinsufficiency, the hallmark of A20, was found. This variant is not yet described in the Infever database (5). *In silico* prediction tools predict it as a disease-causing mutation. Our patient responded well to colchicine and so far did not need additional treatment. Growth and development are so far normal.

## 6. Conclusion

In the present case report, we wanted to emphasize the importance of genetic testing in case of an unclear clinical picture. The cooperation between clinicians of different specialties and geneticists is of extreme importance in such cases. It is important to find a diagnosis in a child with recurrent fever and other signs of immune dysregulation. Proper treatment, if available, can be offered when the diagnosis is made.

## Data Availability

The datasets for this article are not publicly available due to concerns regarding participant/patient anonymity. Requests to access the datasets should be directed to the corresponding author.

## References

[1] Zhou Q Wang H Schwartz DM Stoffels M Park YH Zhang Y Loss-of-function mutation in TNFAIP3 leading to A20 haploinsufficiency cause an early-onset autoinflammatory disease. Nat Genet. (2016) 48(1):67–73. 10.1038/ng.3459 26642243 PMC4777523

[2] Aksentijevich I Zhou Q. NF-*κ*B Pathway in autoinflammatory diseases: dysregulation of protein modifications by ubiquitin defines a new category of autoinflammatory diseases. Front Immunol. (2017) 8:399. 10.3389/fimmu.2017.00399 28469620 PMC5395695

[3] Aeshlimann FA Batu ED Canna SW Go E Gul A Zhang Y A20 haploinsufficiency (HA20): clinical phenotypes and disease course of patients with a newly recognised NF-kB-mediated autoinflammatory disease. Ann Rheum Dis. (2018) 77(5):728–35. 10.1136/annrheumdis-2017-212403 29317407

[4] Aeschlimann FA Laxer RM. Haploinsufficiency of A20 and other paediatric inflammatory disorders with mucosal involvement. CurrOpin Rheumatol. (2018) 30(5):506–13. 10.1097/BOR.0000000000000532 29916847

[5] https://infevers.umai-montpellier.fr/web/.

[6] Yu MP Xu XS Zhou Q Deuitch N Lu MP. Haploinsufficiency of A20 (HA20): updates on the genetics, phenotype, pathogenesis and treatment. World J Pediatr. (2020) 16(6):575–84. 10.1007/s12519-019-00288-6 31587140

[7] Wu Z Zheng Y Sheng J Han Y Yang Y Pan H CD3^+^ CD4^−^CD8^−^ (double-negative) T cells in inflammation, immune disorders and cancer. Front Immunol. (2022) 13:816005. 10.3389/fimmu.2022.816005 35222392 PMC8866817

[8] Liu J Lin Y Li X Ba H He X Peng H Haploinsufficiency of A20 in a Chinese child caused by loss-of-function mutations in TNFAIP3: a case report and review of the literature. Front Pediatr. (2023) 10(10):990008. 10.3389/fped.2022.990008 36727002 PMC9885370

[9] Schwartz DM Blackstone SA Sampaio-Moura N Rosenzweig S Burma AM Zhang Y Type I interferon signature predicts response to JAK inhibition in haploinsufficiency of A20. Ann Rheum Dis. (2020) 79(3):429–31. 10.1136/annrheumdis-2019-215918 31767699 PMC7507120

